# Recommendations for Sustainable Ancient DNA Research in the Global South: Voices From a New Generation of Paleogenomicists

**DOI:** 10.3389/fgene.2022.880170

**Published:** 2022-04-26

**Authors:** Maria C. Ávila-Arcos, Constanza de la Fuente Castro, Maria A. Nieves-Colón, Maanasa Raghavan

**Affiliations:** ^1^ International Laboratory for Human Genome Research, Universidad Nacional Autónoma de México, Querétaro, Mexico; ^2^ Department of Human Genetics, University of Chicago, Chicago, IL, United States; ^3^ Department of Anthropology, University of Minnesota Twin Cities, Minneapolis, MN, United States

**Keywords:** paleogenetics, sustainable research, regulation, global south, diversity, accountability

## Abstract

Paleogenomics - the study of ancient genomes - has made significant contributions, especially to our understanding of the evolutionary history of humans. This knowledge influx has been a direct result of the coupling of next-generation sequencing with improved methods for DNA recovery and analysis of ancient samples. The appeal of ancient DNA studies in the popular media coupled with the trend for such work to be published in “high impact” journals has driven the amassing of ancestral human remains from global collections, often with limited to no engagement or involvement of local researchers and communities. This practice in the paleogenomics literature has led to limited representation of researchers from the Global South at the research design and subsequent stages. Additionally, Indigenous and descendant communities are often alienated from popular and academic narratives that both involve and impact them, sometimes adversely. While some countries have safeguards against ‘helicopter science’, such as federally regulated measures to protect their biocultural heritage, there is variable oversight in others with regard to sampling and exportation of human remains for destructive research, and differing requirements for accountability or consultation with local researchers and communities. These disparities reveal stark contrasts and gaps in regional policies that lend themselves to persistent colonial practices. While essential critiques and conversations in this sphere are taking place, these are primarily guided through the lens of US-based heritage legislation such as the Native American Graves and Protection Act (NAGPRA). In this article, we aim to expand the scope of ongoing conversations by taking into account diverse regional contexts and challenges drawing from our own research experiences in the field of paleogenomics. We emphasize that true collaborations involve knowledge sharing, capacity building, mutual respect, and equitable participation, all of which take time and the implementation of sustainable research methods; amass-and-publish strategy is simply incompatible with this ethos.

## Introduction

The field of ancient DNA (aDNA) has grown from a marginal subject that faced early scepticism, to a highly attractive and expanding field that has produced significant knowledge on the evolutionary history of several species, especially our own (Orlando et al., 2021). Numerous methodological improvements, including next generation sequencing, have considerably increased the yield of authentic aDNA from degraded biological materials. This is advantageous because aDNA research requires the destruction of valuable tissues or materials, making it imperative to handle this limited resource ethically, with legitimate justification, and to secure the recovery of as much information as possible from small sample sizes.

A breakthrough in human aDNA research was the development of “capture” technology, characterizing a subset of approximately 1,240,000 genome-wide variant sites (’1240k’ panel) that are primarily informative for population history inference. Notably, the capture approach was conceptually proposed in 2014 as a strategy to “democratize” the field by offering a cost-effective alternative to whole-genome/shotgun sequencing (Pickrell and Reich, 2014). However, for commercial reasons, this capture assay was initially not publicly accessible to all research groups, which ironically did the opposite of democratization by concentrating the power of this method to groups collaborating with its developers and early adopters.

The cost benefits of this method incited the few research groups with access to this technology and those with large budgetary resources to seek and stockpile ancient human samples. The flames of this “bone rush” (Fox and Hawks, 2019) were further fanned by the sensationalization of aDNA findings via “high impact” publications and popular media, raising a number of ethical concerns (Lewis-Kraus, 2019). Many of these sample collection campaigns were initially done without strong scrutiny by the scientific community. However, in recent years, critics have raised concerns about this “grab-and-go” approach, calling out its extractive nature and lack of engagement or meaningful involvement with local researchers and communities (e.g. (Bardill et al., 2018; Claw et al., 2018; Hudson et al., 2020; Tsosie et al., 2020; Wagner et al., 2020; Argüelles et al., 2022), more references in ^1^). This practice is particularly conflictive when involving the collection and destruction of samples from nations in the “Global South”–a term often used to identify lower-income countries, many of which have been historically oppressed by colonialism (Dados and Connell, 2012)–by laboratories in the “Global North” (the complementary set of countries, many of which earned their higher wealth by colonization and exploitation of “Global South” nations).

Most criticisms of such unethical practices in paleogenomics research focus on United States contexts, the Native American Graves and Protection Act (NAGPRA) and Indigenous rights (Claw et al., 2018; Fox and Hawks, 2019; Wagner et al., 2020). Although a piece recommending ‘global guidelines’ for aDNA research was recently published, it was received with concern by some academics (Somel et al., 2021; Tsosie et al., 2021). As aDNA researchers from, and doing research in, Global South regions, we aim at expanding the discussion further by drawing from our own experiences to contribute to the ethical development of the paleogenomics field in Chile, India, Mexico, and Puerto Rico. We focus on four common challenges faced in our efforts to develop aDNA research programs anew as well as partner with existing programs in these places: 1) Cultural and heritage management regulations, 2) Local funding and infrastructure, 3) Local research and training capacity building, and 4) Consultation with Indigenous and descendant communities. We discuss these issues in the context of the aforementioned countries and outline recommendations from our experiences to address them, though admittedly not the only strategies for doing so.

In writing this piece, we acknowledge our positionality as researchers who may or may not share the same identities and histories with the communities with which we collaborate. We also recognize that our privileges as scientists impact our work and access to resources. The perspectives we discuss here represent our ongoing efforts as we learn, together with our community partners and trainees, how to build more sustainable, fair, and representative aDNA research programs globally.

### Cultural and Heritage Management Regulations

Regulations surrounding destructive sampling and sample export for aDNA research projects vary widely and are enforced at different levels within governmental cultural or heritage institutions (Marquez-Grant and Fibiger, 2013). Regulatory bodies within these institutions are usually tasked with evaluating formal written requests to access collections and perform sampling and assure compliance with final reporting requirements. Besides institutions that enforce federal and/or state heritage regulations, in countries such as the United States and Canada, some Indigenous communities have their own regulations for genetic research studies (Claw et al., 2018, 2021; Begay et al., 2019; Wagner et al., 2020).

However, the reality in the Global South can be very different. Institutions tasked with regulating access to heritage or archaeological collections may lack or have unclear regulations and requirements, insufficient budgets or enforcement strategies to enact these regulations, or a combination thereof (e.g. (Abarca Labra et al., 2018)). This ambiguity can also affect local institutions or individuals (e.g. archaeologists leading excavations) who first receive research proposals for destructive analysis and where the sampling process itself may not be clearly defined nor accountability or follow-up measures outlined, leaving important decisions about sampling and research practices in the hands of a few.

Within Latin America, there is large variation in regulations for destructive sampling and few that are particularly dedicated to aDNA. For example, both Mexico and Chile have specific institutions dedicated to the research, conservation and protection of anthropological, archaeological, historical, and cultural heritage (National Institute of Anthropology and History (INAH) and National Monuments Council, respectively). INAH’s Archaeology Council regulates access to archaeological samples for all destructive and non-destructive analyses following institutional guidelines (Instituto Nacional de Antropología e Historia, 2019). Although the council has clear requirements for ancient bio-molecular research, some of them are not ready to be implemented (e.g., lack of infrastructure to store aDNA extracts, libraries, or genetic data). In some countries from this region, cultural artifacts and human bodies recovered from archaeological contexts are subject to a heritage process where there is a centralized entity managing their fate. This process primarily involves legal protection against destruction and variable levels of regulations for research settings, particularly if samples are leaving the country. Albeit necessary, there are some troubling assumptions under this model that are rarely discussed by aDNA researchers, particularly in relation to regulatory and state recognition of Indigenous identities, which we discuss in following sections.

In India, the Archaeological Survey of India (ASI) serves as the premier national governmental institution that oversees the cultural heritage of the country, including regulating the export of materials abroad for research. Additionally, state government archaeological departments have the autonomy to carry out the role of heritage conservation and conduct archaeological excavations in their respective jurisdictions. These institutions are well suited to implement and oversee a best-practice regulatory framework for sampling of human bodies from archaeological contexts for aDNA research (Jamir, 2022b; Pappu, 2022; Rai, 2022; Taher, 2022).

Historically unequal power relations can also shape heritage management in ways that have consequences for aDNA research as seen in Puerto Rico, which as a U.S. territory without federally recognized Indigenous nations, is excluded from NAGPRA (Rodríguez López, 2009b; d’Alpoim Guedes et al., 2021). While artifacts and human bodies recovered from archaeological contexts are considered the patrimony of all Puerto Ricans under local legislation, such laws are superseded by federal regulations (Pagán-Jiménezand Rodriguez Ramos, 2008; Rodríguez López, 2009a;, 2009b; Llorens-Liboy and Núñez, 2011). Indeed, many archaeological remains excavated in Puerto Rico were exported to the US mainland soon after the American invasion (Pagán Jiménez, 2000); well before Puerto Ricans could vote for their own government or enact modern heritage legislation. Now curated in museums and collections abroad (DaRos and Colten, 2009; Françozo and Strecker, 2017), these remains can be legally sampled for aDNA research without passing through the permitting process required by Puerto Rican authorities or consulting with island stakeholder communities.

Recommendation: Support efforts to improve local cultural and heritage management regulations and involve these institutions in the research process. Researchers can aid communities, permitting agencies, and ethical and regulatory boards seeking to develop better frameworks and guidelines for destructive aDNA analyses by holding open discussions with board members about the process, risks and potential benefits of aDNA research, and by providing detailed reports and inventories of the remaining DNA products and data files after research is concluded. Furthermore, engaging with local museums or other heritage management institutions may facilitate contacts with communities for consultations prior to study start and assist with dissemination of research results to the general public, local museums, universities or schools.

### Local Research and Training Capacity Building

Although building local capacities for research is an essential first step towards ensuring sustainability, equity and inclusion within paleogenomics, many Global South nations face significant challenges in setting up training programs and maintaining research facilities. These challenges include economic austerity measures that underfund public education and scientific investment, small or nonexistent research funding streams, lack of support for research capacity building, and limited access to, or structural disparities in, higher education (Reidpath and Allotey, 2019; Reidpath and Allotey, 2020; Viera et al., 2020; Carter and Hujo, 2021).

In Latin America and the Caribbean, for example, enrollments in higher education have increased over the last decade but other inequities persist, such as patterned access to higher education based on income and low availability of coursework or degree programs in science fields (Ferreyra et al., 2017). For example, despite being a US territory, Puerto Rico’s universities are under-funded and about 42.9% of undergraduate students live below the poverty line (Nazario, 2014; Trines, 2018). As of this writing, there are limited opportunities for local undergraduate training in anthropology (Pagán Jiménez, 2000; Pagán-Jiménezand Rodriguez Ramos, 2008), and no formal graduate degree programs focused on biological anthropology, bioarchaeology, ancient DNA, or genomic science. While such programs can be pursued abroad, they often charge high tuition rates, making them unaffordable for many families, or limiting access just to high-income students. In Mexico, where a few degree programs in anthropology and genomics provide training in paleogenomics methods, severe budget cuts and government divestment threaten to reduce offerings and shutter educational institutions (Santos Cid, 2022). In India and Chile, degree programs in anthropology, archaeology, and genomics exist; however, there is currently only one functional aDNA lab in each country to train and conduct all local paleogenomics research.

Altogether, these factors make it difficult for local students to access and complete degree programs, reduce local job opportunities for scientific professionals, and accelerate brain-drain emigration patterns (Mishra, 2006; Weinberg, 2011; Docquier, 2014). In such contexts, local students may understandably see Global North countries as the only options for training, and local researchers may choose to export aDNA samples to these locations for processing and analyses. However, this creates a vicious cycle, as export means no local research is conducted, no training of students takes place, and local capacity for research remains underdeveloped.

Recommendation: Provide training opportunities to strengthen local research capacity. Researchers from Global North institutions can fund bilateral trainee exchanges with local institutions and provide wet lab and bioinformatics training in their labs. As shown by the success of the SING consortium workshops in the United states, Canada, Australia and Aotearoa (Malhi and Bader, 2015; Wade, 2018), short courses and training events can also become spaces to discuss multiple aspects of genomics research, including sampling strategies, data analyses or interpretations, and ethical considerations with local students and community members.

### Local Funding and Infrastructure

Ancient DNA research requires sterile conditions for data generation and high data quality standards. This calls for an entirely separate facility dedicated to low copy number DNA processing that complies with strict criteria (Knapp et al., 2012; Fulton and Shapiro, 2019), and uses sterile molecular grade reagents and consumables (Champlot et al., 2010; Llamas et al., 2017). In the last 5–6 years, paleogenomics data generation has been overtaken by the ‘1240k’ capture panel, costing nearly $250 per reaction without accounting for shipping and other export regulatory costs. Despite its early branding as “democratizing” paleogenomics (Pickrell and Reich, 2014), the capture panel remains out of reach for many laboratories in the Global South and may become yet another means of unequitable foreign collaborations (Argüelles et al., 2022).

Altogether, the cost of establishing, maintaining, and day-to-day running of a paleogenomics facility is not an easy feat, often requiring institutional and/or governmental commitment to infrastructure (e.g., lab space, equipment) and sustenance (e.g., hiring, reagents, consumables, maintenance). Not surprisingly, paleogenomics is not a high priority research avenue in most countries battling more pressing challenges, such as health crises or economic insecurity (Maher et al., 2012; Lebel and McLean, 2018; Liverpool, 2021). This often translates to little to no support for local researchers interested in developing this field in their countries, as reflected in the current distribution of global aDNA laboratories^2^. For countries in the Global South that have aDNA laboratories, upkeep is often difficult with issues ranging from power shortages to infrastructural and maintenance limitations such as limited space and scope for expansion, delayed and pricey access to equipment and parts, lack of expertise to diagnose and repair breakdown of proprietary equipment, and so on. Moreover, sourcing and ordering reagents that are easily obtained in the Global North is time-consuming and often several fold more expensive elsewhere (Table 1) (Ciocca and Delgado, 2017; Valenzuela-Toro and Viglino, 2021). When adding publication and conference fees, and the US dollar advantage, the cost of conducting research becomes disproportionately higher in the Global South vis-à-vis available budgets. Ultimately, these disparities in resource availability for executing scientific programs creates opportunities for ‘grab-and-go’ strategies that further research inequity and helicopter science practices (Adame, 2021; Haelewaters et al., 2021).

**TABLE 1:** Comparison of costs and delivery times for select consumables and reagents commonly used in aDNA research involving genome-wide next-generation sequencing. Cost estimates include tax, customs duties and shipping fees. Delivery times are the average number of days between ordering and arrival.

Country	Chile		India		Mexico		United States (UChicago)	
Reagent/Consumable	Cost (USD)	Time (days)	Cost (USD)	Time (days)	Cost (USD)	Time (days)	Cost (USD)	Time (days)
Filter tips (one set of P2/P10,P100/P200 and P1000)	$30	7	$75-$100	30–60	$29	14–60	$17	7
Sequencing (cost per Gb on the platform used most by local aDNA facility)	NextSeq550 2 × 75 (18 Gb); $100	30	HiSeq 4000 (120 Gb); $25	30–60	NextSeq550 2 × 75 (18 Gb); $82	21–30	NovaSeq SP 1 × 100 (45 Gb); $38	7–14
dsDNA library reagents; 1. NEBNext End Repair (E6050S); 2. NEBNext Quick Ligation E6056S; 3. Bst Polymerase Large Fragment M0275A	1. $172;2. $607;3. $137	14–21	1. $100;2. $260;3. $100	120–180	1. $196;2. $624;3. $132	20–30	1. $74;2. $262;3. $59	7

Recommendation: Foster equitable collaborations by supporting local research capacity and involving knowledgeable local collaborators and researchers as equal partners at all stages. Researchers based at Global North institutions can do this at different levels, from sharing equipment and reagents to local laboratories to formally collaborating with local researchers on projects and international grants. If collaborating in countries that already have aDNA facilities, additional ways to support capacity are to write research grants together with local collaborators, and budget for both reagents and consumables to be ordered to the local lab and travel to process samples jointly in the local laboratory and plans to contribute to training. The expertise and experience of local researchers and institutions can contribute nuanced insights and guide research goals to focus on locally relevant questions. Local collaborators are likely to have a better understanding of the regional history, current socio-political situation, regulations and, importantly, ethical implications of research for local communities. Partnerships with local collaborators and institutions can also aid in developing culturally responsive materials (Judd and McKinnon, 2021), in their language, for dissemination of research results to the public via news sources or other venues. This is important as genetics papers are often loaded with scientific jargon and assumptions that may be hard to interpret and open to misinterpretation by those far from the research (Harmon, 2018; Reich, 2018; Gannon, 2019; Wolinsky, 2019; Panofsky et al., 2021).

### Consultation with Indigenous and Descendant Communities

In the United States, NAGPRA legally requires researchers to identify affiliated tribal nations for consultation about research with ancestral remains. Meanwhile, in many Global South countries, in addition to the lack of legal mandates for community consultation, there are other issues surrounding unclear regulations on heritage management (discussed above), Indigenous identities, and heterogeneity in the state-Indigenous dynamics across and within regions that should be considered while enacting nuanced, community-sensitive consultation and engagement strategies.

Ethnic identity and belonging are fluid sociocultural constructs with definitions that vary over time and between populations. In some Indigenous communities, these constructs can be tied to connections with land or ancestors, and with spiritual, cultural, religious and linguistic traits, while others may invoke biological (phenotypic) features. Insights from genetic ancestry studies, if not framed sensitively and acknowledging existing identity structures, could impose upon the process of identity construction for both individuals and communities (Egorova, 2009; Gibbon et al., 2011; TallBear, 2013; Wade et al., 2015; Benn Torres and Torres Colón, 2020; Crellin and Harris, 2020). Genetic insights can sometimes conflict with community and individual beliefs or reproduce nationalistic or essentialized notions of identity that suppress the existence of Indigenous peoples.

In some Latin American countries, including Mexico and Chile, the institutional de-indigenization processes put in practice by the state emphasize that most of the population is mestizo, trivializing and legally neglecting the inherent value of Indigenous ancestry (Vasconcelos, 1997; García Deister, 2014; Manrique, 2016; Wade, 2017). While Indigenous peoples have some legal recognition in these countries, the discourse of a majority mestizo nation erases Indigenous rights over ancestral lands and heritage under the illusion that all mestizos have equal rights over Pre-Hispanic cultural heritage (Endere and Ayala, 2012; Silva et al., 2022). Afrodescendant communities, who have faced historical marginalization and invisibility in Latin American countries, encounter similar challenges because national identity in these countries is so strongly tied to mestizaje (Arocha and Maya, 2008; Weltman-Cisneros and Tello, 2013; Agren, 2020). To illustrate how this misconception can permeate scholarship, a recent piece on global guidelines for aDNA research (Alpaslan-Roodenberg et al., 2021) wrongfully claimed that mestizos in many Latin American countries “embraced their Indigenous roots”, hence the request to consult with Indigenous peoples in this region was “paternalistic” and “colonialist”. By reproducing such harmful narratives that relegate Indigenous peoples to legacies that should be considered a matter of the past and only embedded in the present-day mestizo national identity, such statements reproduce a long history of institutional discrimination and Indigenous erasure in Latin America. While consultation with Indigenous peoples for aDNA research outside the United States is a complex subject, for which NAGPRA protocols cannot be directly applied, stating that it is not needed because Indigenous identity is well represented by the mestizo population and State institutions is a fallacy and a continuation of historical harms.

In India and other parts of South Asia, the dynamics and recognition of Indigenous identities and rights in heritage management may differ from other places discussed above and even display vast intra-regional heterogeneity but, ultimately, result in a similar undermining of Indigenous engagement and involvement that are, to our knowledge, not currently mandated in archaeological (Jamir, 2022a) or aDNA research.

In this context, from an aDNA researcher’s perspective, identifying Indigenous populations that could be affiliated to individuals found in a given archaeological site or museum collection is not straightforward. In places like Mexico, where ancient empires like the Mexica invaded many territories and where multiple cultures could coexist in a single place (Mata-Míguez et al., 2012; Manzanilla, 2017), an additional layer of complexity emerges. Even if these connections can be made, challenges can surface if present-day Indigenous communities do not hold spiritual affiliation to ancestors from many generations ago (Cucina, 2013; Whittaker, 2020), if they have never been consulted about research participation before or face other more immediate challenges to their sovereignty or livelihoods (Castellanos, 2020; Hesketh, 2021; Rodriguez Aguilera, 2021). Because of the high levels of poverty and injustice most Indigenous communities in the Global South face (Hall and Patrinos, 2012; Hall and Gandolfo, 2016), an aDNA researcher can be hesitant of whether bringing yet another issue to consult and decide upon (especially one that has never been considered before) is correct or if it is invasive or imposed.

Recommendation: Prioritize community engagement as an integral part of the research design. As outlined above and elsewhere (Wagner et al., 2020), there is no one-size-fits-all set of guidelines. Instead of foregoing the engagement process entirely because of the inherent complexity, the subject of community consultation needs to be discussed thoroughly and applied to each circumstance, while including Indigenous scholars and other stakeholders in deciding the most ethical approach for each place, regardless of whether local legislation requires consultation or Indigenous approval to carry out the project. This requires dedicated time and resources to research the history and present situation of the region and communities one wants to work with, and then preparing to engage with and involve them in horizontal discussion before, during and after the project. Integrating descendant community perspectives into research should not be seen as a burden or checkboxing an outreach step (Muller and Dortch, 2020). Instead, such discussions acknowledge and respect the richness of community-based knowledge that can additionally significantly enrich the research process (Wagner et al., 2020).

## Conclusion

To conclude, we propose applying a “glocal” approach to aDNA research in the Global South. The glocal principle highlights the importance of assessing global-local interactions by considering the local context within a coherent global pattern. As described by (Patton, 2020), global systems must be contextually sensitive and grounded in the interaction between local and global processes. In aDNA research, this would entail applying global premises of sustainability and justice and maintaining awareness of the historical harms caused by scientific colonialism, extractivism, and other forms of exploitation of Global South nations by Global North researchers (Argüelles et al., 2022). Locally, aDNA researchers must be attuned to the implications of their research, especially regarding heritage regulation and management, knowledge and resource sharing, the development or strengthening of local expertise, involvement of Indigenous communities, and conflicts that may arise with traditional knowledge systems. More broadly, institutions in the Global North, such as funding agencies and academic promotion and tenure committees, can support efforts that prioritize community engagement by recognizing or funding this work as an integral component of the research process. Importantly, we strongly believe that for the field of aDNA to meet these ethical responsibilities, the pace must not be dictated by the growth of the field (Alpaslan-Roodenberg et al., 2021), but by prioritizing the requisite time to build and implement accountability measures. Despite the time and effort required, we find that such commitments also foster the creation and maintenance of long-term partnerships which can ultimately aid the research process and lead to a more sustainable, just and inclusive paleogenomics research field for the Global South.

## Data Availability

The original contributions presented in the study are included in the article/Supplementary Material, further inquiries can be directed to the corresponding authors.

## Footnotes

1
https://elsihub.org/index.php/collection/game-bones-power-ethics-and-emerging-technology-paleogenomics-research.

2
https://www.google.com/maps/d/u/0/viewer?mid=1qwXOKV5uoQntgBsxQrxS01YHpbs&ll=52.19802207086798%2C-5.195632878906142&z=5 and https://isogg.org/wiki/List_of_forensic_and_ancient_DNA_laboratories.
